# Attitudes towards Maternal Immunisation of Polish Mothers: A Cross-Sectional, Non-Representative Study

**DOI:** 10.3390/vaccines12101143

**Published:** 2024-10-05

**Authors:** Iwona Kiersnowska, Kinga Kalita-Kurzyńska, Weronika Piekutowska-Kowal, Joanna Baranowska, Edyta Krzych-Fałta

**Affiliations:** 1 Department Basic of Nursing, Faculty of Health Sciences, Medical University of Warsaw, 27 Erazma Ciołka St., 01-445 Warsaw, Poland; iwona.kiersnowska@wum.edu.pl (I.K.); zpp@wum.edu.pl (W.P.-K.); edyta.krzych-falta@wum.edu.pl (E.K.-F.); 2 Department of Medical Biology, Medical University of Warsaw, 14/16 Litewska St., 00-575 Warsaw, Poland; 3 St Sophia’s Specialist Hospital, 90 Żelazna St., 01-004 Warsaw, Poland; pol.joannabaranowska@gmail.com; 4 Institute of Sexuology and Psychotherapy, 25 Nowogrodzka St., 00-511 Warsaw, Poland

**Keywords:** maternal immunisation, vaccinations, pregnancy, attitude, VAX Scale

## Abstract

Introduction: Vaccination protects pregnant women against dangerous infectious diseases and contributes to disease prevention for the child until their vaccination schedule begins. Vaccination behaviour is related to attitudes concerning vaccine prevention. Materials and Methods: This cross-sectional, non-representative study was conducted among Polish mothers using a diagnostic survey. We used the Vaccination Attitudes Examination (VAX) Scale. The author’s questionnaire included questions concerning experiences with adult and maternal vaccinations, as well as sociodemographic data. Results: Among the 375 respondents, more than half (n = 208, 55.47%) received at least one vaccine during pregnancy. The majority of respondents had a university education (n = 356, 94.93%). There was no statistically significant difference in terms of receiving vaccines during pregnancy between respondents with and without a healthcare education (*p* = 0.230). A logistic regression model indicated that women who were vaccinated outside pregnancy for COVID-19 (OR 4.61, 2.60–8.22) and influenza (OR 7.14, 3.58–14.25) were statistically significantly more likely to be vaccinated during pregnancy. There were statistically significant differences between women who were vaccinated during pregnancy and those who did not receive maternal immunisation in three subscales of the VAX Scale: *Mistrust of Vaccine Benefit*, *Concerns about Commercial Profiteering*, and *Preference for Natural Immunity* (*p* < 0.001). The most frequently indicated reason for a woman’s decision to be vaccinated was concern for the health and safety of the unborn child (n = 196, 94.23%). In contrast, women who were not vaccinated reported fear for the health and safety of the unborn child (n = 88, 52.69%). Conclusions: A key point may be to contribute to the vaccinology education of healthcare professionals, who play an important role in pregnant women’s decision to be vaccinated. However, our results may be disrupted because the majority of the women in our study had a university education.

## 1. Introduction

Vaccinations support the immune system by helping the body’s natural defence mechanisms. They not only reduce the risk of disease but also the number of disease-related complications, and they prevent 3.5–5 million deaths per year [1]. Maternal immunisation has been recommended by the World Health Organization (WHO) since 2005. Studies have shown that vaccine prevention during pregnancy is safe for both the mother and child. Vaccination protects women against dangerous infectious diseases. There is also the additional benefit of antibodies transferring across the placental barrier and breast milk, resulting in immunisation of the newborn. This contributes to disease prevention until the child’s vaccination schedule begins [2,3,4].

Vaccination behaviour is related to attitudes concerning vaccine prevention. Identifying areas of doubt can help us to understand these behaviours [5]. Pregnant women are also guided by the perceived risks (risk of perceived vaccine harm and known side effects) and benefits (protection of the pregnant woman, foetus, and baby) when deciding whether to be vaccinated [6]. Studies indicate that recommendations by healthcare professionals play a major role in whether women decide to be vaccinated during pregnancy [6,7].

In most European countries, the guidelines for maternal immunisation include vaccinations against influenza and pertussis. From 2021, recommendations expanded to include the COVID-19 vaccination [8,9]. In Poland, vaccine prevention against seasonal influenza and diphtheria, tetanus, and pertussis (Tdap) has been recommended for pregnant women for many years. For the influenza vaccination, an inactivated vaccine is recommended which is administered seasonally between September and March. It can be given in any trimester of pregnancy. Tdap vaccination is recommended between 27 and 36 weeks of pregnancy. In April 2021, the Polish Society of Gynaecologists and Obstetricians recommended COVID-19 vaccination during pregnancy. Since then, mRNA vaccination has been recommended for pregnant women and can be given anytime during pregnancy. However, a full vaccination course (i.e., two doses) should be completed before the beginning of the third trimester [10,11,12]. Maternal immunisation, like other vaccinations recommended in Poland, is paid for by the patient; however, the pregnant woman does not cover the cost of qualifying for the vaccination [13].

Due to the fact that vaccine prevention for pregnant women is epidemiologically relevant, this study focuses on attitudes and factors influencing women’s decisions about maternal vaccinations against COVID-19, influenza, and diphtheria, tetanus, and pertussis. We had difficulty obtaining a representative sample of respondents in terms of educational level in our study.

## 2. Materials and Methods

### 2.1. Study Design and Setting

This cross-sectional, non-representative study was conducted among Polish mothers using a diagnostic survey.

### 2.2. Data Collection

We used two study tools: the author’s questionnaire and the Vaccination Attitudes Examination (VAX) Scale.

The author’s questionnaire included questions concerning experiences with adult and maternal vaccinations as well as sociodemographic data. In the context of maternal vaccinations, we focused on the kinds of vaccines received, motivations towards maternal vaccine prevention, and reasons to remain unvaccinated during pregnancy.

The VAX Scale was designed and validated by L.R. Martin and K.J. Petrie. It constitutes a 12-item scale that is short and has high internal consistency reliability. It consists of four subscales, including the following correlated factors: *Mistrust of Vaccine Benefit*, *Worries about Unforeseen Future Effects*, *Concerns about Commercial Profiteering*, and *Preference for Natural Immunity*. Each factor consists of answers to four questions using a 6-point Likert-type scale ranging from “Strongly disagree” to “Strongly agree” [5]. In this study, we used the VAX Scale, which was translated into Polish by J. Borchet, M. Iwanowska, K. Bałandynowicz-Panfil, and M. Łosiewicz [14]. The internal consistency of the VAX Scale was evaluated using Cronbach’s α, with values of 0.83 in *Mistrust of Vaccine Benefit*, 0.73 in *Worries about Unforeseen Future Effects*, 0.90 in *Concerns about Commercial Profiteering*, and 0.73 in *Preference for Natural Immunity*. All-scale internal consistency was 0.91.

### 2.3. Sample Size

Assuming a fraction size of 0.6 and a 5% margin of error, the study group size was set at 364. The population was determined based on the number of births after 36 weeks of pregnancy in Poland in the year 2022. In addition, we determined the size of the fraction (60%) on the basis of the Polish survey on the vaccination of pregnant women [15,16].

### 2.4. Participants and Recruitment

This study included postpartum women. The study group was selected based on the following inclusion criteria: women over 18 years of age, had writing and reading skills in Polish, were residing in Poland during their pregnancy, and gave birth after 1 May 2021 (after the adoption of guidelines concerning the COVID-19 vaccinations in Poland). Due to the fact that the Tdap vaccination is recommended in the third trimester, we adopted the criterion of at least 34 weeks of gestation at the date of delivery.

Participant recruitment was carried out using a computer-assisted web interview (CAWI) that was prepared using Google Forms. Information about the study was communicated directly to potential respondents by providing a description of the study’s brief and a link to the questionnaire on social media in mother’s groups and groups for nurses/midwives on Facebook and Instagram. All respondents were informed that participation in the survey was anonymous and voluntary.

### 2.5. Statistical Methods

The level of statistical significance was *p* < 0.05. After establishing the lack of normal distribution using the Shapiro–Wilk test, quantitative variables were compared using the Mann–Whitney U test. Qualitative variables were compared using the chi-square test. Correlations between variables were examined using Spearman’s test. Multivariate regression models were built to examine the effect of receiving vaccines on domain-specific beliefs using the variables immunisation during pregnancy and inoculation with individual vaccinations.

The examined power of the tests was >0.90 for our analysis. The calculations were performed using Statistica 13.1. (StatSoft Poland, Krakow, Poland).

### 2.6. Ethical Considerations

Participation in this study was voluntary and anonymous. This study was conducted according to the principles of the Declaration of Helsinki and reported to the Bioethical Committee of the Medical University of Warsaw, Poland (approval number: 29/2024).

## 3. Results

### 3.1. Participants

**Figure 1:**
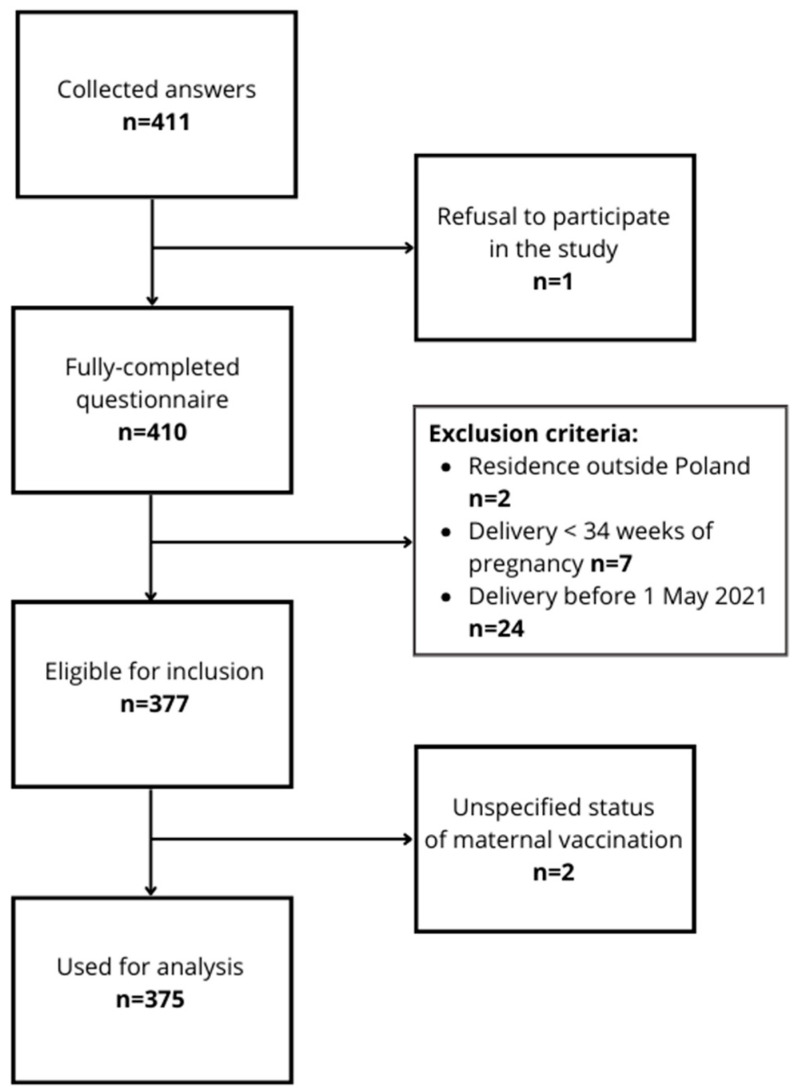
Flowchart of the study recruitment process.

We collected 411 answers. Due to refusal to participate in this study or not meeting the inclusion criteria, 38 questionnaires were excluded from the statistical analysis, leaving 375 participants in total (Figure 1).

### 3.2. Descriptive Data

Table 1 shows the study group’s characteristics. The study participants were women from all over Poland. The ages of the respondents ranged from 22 to 45 years (average age: 32.32 ± 4.22 years, median age: 32 years). The majority of the participants were residing in cities with over 500,000 inhabitants (n = 206, 69.33%) and had a university education (n = 356, 94.93%). Almost one-third of the women reported that their education was related to healthcare, which included nurses, midwives, and doctors (n = 116, 30.93%).

### 3.3. Main Results

More than half (n = 208, 55.47%) of the study participants received at least one vaccine during pregnancy. Among the vaccinated women, the most common maternal vaccination was that against diphtheria, tetanus, and pertussis (n = 166, 79.81%). Almost half of these respondents were vaccinated against COVID-19 (n = 97, 46.63%), and more than one-third received the influenza vaccine (n = 77, 37.02%). 

#### 3.3.1. Vaccine Prevention outside the Pregnancy Period and Use of Maternal Vaccinations

An age-corrected logistic regression model indicated that women who were vaccinated outside pregnancy for COVID-19 and influenza were statistically significantly more likely to be vaccinated during pregnancy. Detailed data are included in Table 2.

#### 3.3.2. Study Participants’ Healthcare Education and Use of Maternal Vaccinations

Table 3 shows the maternal vaccination use in the latest pregnancy among the study participants, depending on their education. There was no statistically significant difference in receiving vaccines during pregnancy between the respondents with and those without a healthcare education (*p* = 0.230). Also, there were no statistically significant differences in the COVID-19 (*p* = 0.620) or influenza (*p* = 0.902) vaccinations given outside the pregnancy period according to the study participants and their education (Table A1).

#### 3.3.3. Attitudes towards Vaccine Prevention

The particpants’ attitudes towards vaccination were assessed based on the VAX Scale. There was no statistically significant correlation between the surveyed women’s ages and their attitudes in each subscale of the VAX Scale (rho < 0.250, *p* > 0.05). Similarly, the respondents did not differ statistically significantly in their attitudes across domains in terms of level of education (rho < 0.160, *p* > 0.05).

There were statistically significant differences between the women who were vaccinated during pregnancy and those who did not receive maternal vaccine prevention in three subscales of vaccination attitudes: *Mistrust of Vaccine Benefit*, *Concerns about Commercial Profiteering*, and *Preference for Natural Immunity* (*p* < 0.001) (Table 4). 

We used multiple regression to build models explaining the impact of being vaccinated during pregnancy on the women’s attitudes in each subscale of the VAX Scale. Maternal vaccination had an influence on the lower scores in the domains *Mistrust of Vaccine Benefit, Concerns about Commercial Profiteering*, and *Preference for Natural Immunity* (*p* < 0.001). No change was observed in *Worries over Unforeseen Future Effects* (*p* = 0.751) (Table 5).

We used multiple regression to build models explaining the impact of receiving specific vaccines during pregnancy on the women’s attitudes in each subscale of the VAX Scale. Maternal vaccination against diphtheria, tetanus, and pertussis was statistically significant in reducing women’s scores for *Mistrust of Vaccine Benefit*, *Concerns about Commercial Profiteering*, and *Preference for Natural Immunity* (*p* < 0.001).

In contrast, in women vaccinated against COVID-19 during pregnancy, there were statistically significant limitations in the following domains: *Mistrust of Vaccine Benefit*, *Concerns about Commercial Profiteering*, and *Preference for Natural Immunity* (*p* < 0.05). However, an increase in *Worries over Unforeseen Future Effects* was observed (*p* < 0.001) (Table 6).

#### 3.3.4. Reasons for Use or Non-Use of Maternal Vaccine Prevention

The most frequently indicated reasons for a woman’s decision to be vaccinated during pregnancy were concern for the health and safety of the unborn child (n = 196, 94.23%), concern for their own health and safety (n = 139, 66.83%), and recommendations from the pregnant woman’s healthcare provider (*p* = 135, 64.90%). Detailed information is shown in Table 7.

In contrast, women who were not vaccinated during pregnancy reported no recommendations from their healthcare provider (*p* = 103, 61.68%), fear for the health and safety of the unborn child (*p* = 88, 52.69%), and fear for their own health and safety (*p* = 51, 30.54%). Only one woman (0.60%) reported medical contraindications to vaccination during pregnancy (Table 8).

## 4. Discussion

In this study, more than half (n = 208, 55.47%) of the study participants received vaccination(s) during pregnancy, especially against diphtheria, tetanus, and pertussis (79.81% of vaccinated women). The second most common vaccine uptake was against COVID-19 (n = 97, 46.63% of vaccinated women). The least used maternal immunisation by the respondents was the influenza vaccine (n = 77, 37.02% of vaccinated women). Jurga et al. conducted a study among 205 postpartum patients at the Department of Obstetrics and Perinatology of the University Hospital in Cracow, Poland. The majority of these women had received the COVID-19 vaccination (n = 126, 61.5%) during pregnancy. Only a quarter (n = 48, 23.4%) of the respondents had been immunised against diphtheria and pertussis, and only 25 (12.2%) had been immunised against influenza [16].

We observed that, for our study, the study participants who used vaccinations outside pregnancy (for COVID-19 and influenza) received vaccines more frequently during pregnancy (OR = 4.61 and OR = 7.14, respectively). De Brabandere et al., based on a thematic review, indicated that the use of maternal immunisations may have an impact on the acceptance of new vaccines. Their analysis revealed that vaccine uptake against influenza and/or pertussis during pregnancy is a positive determinant for accepting maternal vaccine prevention against COVID-19 [17].

Some researchers have analysed the university education status or professions of their study participants and their attitudes towards immunisation [18,19]. We are not able to relate our results to the educational levels of the respondents, because the majority of them had a university education (n = 356, 94.93%). In our study, healthcare education did not have an impact on the use of maternal vaccinations (*p* = 0.230). Furthermore, our study also concerned adult vaccinations against influenza and COVID-19 (see Table A1). In the survey by Perez et al., among 11,405 pregnancy-capable healthcare workers, 75.3% strongly desired the COVID-19 vaccination, while only 1.5% were very strongly averse to vaccination [20]. In an international study by Lazarus et al., conducted among 23,000 persons from 23 countries, COVID-19 vaccine hesitancy among healthcare workers decreased from 8.1% to 4.6% during the year of the study, and it was significantly lower than in non-healthcare workers [18]. On the other hand, a cross-sectional study by Iguacel et al. among the Spanish general population and workers from university hospitals in three medium-sized cities showed that 1.5% (n = 32) of the respondents rejected the COVID-19 vaccine, but this percentage was higher among healthcare professionals compared to non-healthcare professionals, students, or those who were unemployed [19].

The VAX Scale constitutes a short and simple tool to investigate significant associations regarding vaccination prevention behaviours and intentions. It provides an efficient method for identifying persons with vaccination resistance [5]. It has been readily used by researchers to assess attitudes towards vaccinations [19,21,22,23]. However, few studies using the VAX Scale have focused on pregnant women or maternal vaccine prevention [24,25,26]. Based on the VAX Scale, our respondents who received maternal immunisation had a statistically significantly lower mean score than those who were not vaccinated during pregnancy in two subscales, namely *Mistrust of Vaccine Benefit* (6.02 ± 3.46 vs. 9.82 ± 4.54) and *Concerns about Commercial Profiteering* (5.17 ± 2.55 vs. 8.34 ± 4.09). The mean score for *Preference for Natural Immunity* was statistically significantly higher for the study participants who did not use maternal vaccinations (8.72 ± 4.16 vs. 6.50 ± 2.83). A study by Citu was conducted among Romanian pregnant and non-pregnant women, focusing on vaccines against SARS-CoV-2 [24]. The pregnant respondents demonstrated a more unfavourable approach according to the VAX Scale than the non-pregnant respondents. They scored higher than non-pregnant women in all categories on the VAX Scale. Similar results concerning Pakistani female patients were described in the work of Rehman et al. [25]. The pregnant women from these two studies had higher (or equal) median scores in the subscales *Mistrust of Vaccine Benefit* and *Concerns about Commercial Profiteering* than all our study participants (7 and 8 vs. 6; 6 and 7 vs. 6). The differences in results may be due to the fact that the studies from Romania and Pakistan focused on COVID-19 vaccinations, which have been relatively recently registered on the market. In our study, only women who were SARS-CoV-2-vaccinated during pregnancy showed a statistically significant increase in the subscale *Worries over Unforeseen Future Effects* (*p* < 0.001). Also, those receiving this vaccination had significantly lower scores in the domain *Preference for Natural Immunity* (*p* < 0.001). In contrast, the scores in the domain *Concerns about Commercial Profiteering* were similar for all three types of maternal vaccination.

One of the most important factors influencing vaccine decision-making is pregnant women’s concerns about the safety of vaccinations, which includes fear of adverse events and fear of long-term side effects. The perception of a low risk of disease may also be linked to this factor [27]. However, women’s perceptions of vaccine risk during pregnancy changed significantly throughout the pregnancy. As the pregnancy progressed, women perceived the risk of contracting pertussis to be higher compared to the risk of vaccine side effects [28]. Our study indicates that receiving the vaccination against diphtheria, tetanus, and pertussis during pregnancy has a stronger influence on the limitation of *Mistrust of Vaccine Benefit* (*p* < 0.001) than the uptake of vaccines against seasonal influenza and COVID-19. This may be due to the long-standing recommendation of this vaccination for pregnant women in many countries and consequently higher vaccination coverage.

Healthcare workers can constitute a reliable source of information about maternal immunisation and can influence a patient’s attitudes towards this kind of prevention. Some studies indicate that pregnant women often rely on their medical staff’s knowledge when making a decision regarding vaccination. Health professionals’ recommendations significantly impact patients’ decisions on the use of medications, including vaccines [29]. Recommendations from the pregnant woman’s healthcare provider were the third most common reason to be immunised among our respondents (64.90%, n = 135). At the same time, study participants who were not vaccinated indicated that having no such recommendations was the main reason they were not vaccinated (61.68%, n = 103). Similar results have been obtained by other researchers. According to a study by Napolitano et al., only 9.7% of 372 Italian women received the influenza vaccination because they usually relied on their physician’s advice (88.9%). A lack of information from physicians (34.9%) was the reason most often reported by the respondents for not being vaccinated. Moreover, having a physician’s recommendation about a vaccine was most often cited as contributing to a woman’s positive attitudes towards immunisation [30]. A study by Prospero et al. demonstrated that 95% (n = 348) of Italian women did not get vaccinated against influenza while pregnant. Low adherence to maternal vaccination was associated with having virtually no promotion of vaccination for pregnant women on the part of healthcare workers (OR = 0.16) [31]. A study by Guay et al. showed that vaccine hesitancy was associated with the belief that a healthy lifestyle can eliminate the need for vaccination (aOR = 2.48, 2.09–2.93) and the belief that the use of alternative medicine practices can eliminate the need for vaccination (aOR = 1.39, 1.16–1.68). The next most influential determinants related to vaccine hesitancy were distrust in public health authorities (aOR = 1.40, 1.21–1.63) and perceived insufficient knowledge about immunisation (aOR = 1.26, 1.04–1.51) [32]. 

Studies have shown that pregnant women who seek information about maternal vaccinations and receive information from healthcare professionals are less likely to refuse vaccinations [28]. In addition, in one study, healthcare professionals from 15 countries (n = 1504) indicated that vaccines are important, safe, effective, and compatible with their religious beliefs (99%, 98%, 99%, and 92%, respectively). At the same time, the percentages of surveyed healthcare professionals who felt very comfortable providing explanations to their patients about vaccine safety, the value of vaccines, and the role of adjuvants were 62%, 76%, and 40%, respectively [33].

### Limitations

- There was no demographically representative study group in this study. We did not use random sampling. Respondents with less than a university education represented a small percentage of the study population (n = 19, 5.07%), which resulted in this variable not being included in the models. A study by Montuori et al. pointed out that education level constituted one of the important factors influencing unwillingness/hesitancy to be vaccinated [34];
- We did not include a specific question concerning medical profession. There may be differences between medical professionals. One study showed that nurses and midwives had more concerns about vaccinations than doctors [33].

## 5. Conclusions

There is a need to implement strategies to improve the vaccination rates of pregnant women. A key point may be to contribute to vaccination education among healthcare professionals who, as our study indicates, play an important role in pregnant women’s decisions regarding vaccination. However, our results may be disrupted because the majority of the women who participated had a university education (n = 356, 94.93%).

## Figures and Tables

**Table 1:** Study group’s characteristics (n = 375).

Characteristics		n	%
Level of education	Lower than university education	19	5.07
	University education	356	94.93
Healthcare education	Yes	116	30.93
	No	259	69.07
Place of residence	Village	45	12.00
	City up to 100,000 inhabitants	34	9.07
	City up to 250,000 inhabitants	20	5.33
	City up to 500,000 inhabitants	16	4.27
	City over 500,000 inhabitants	206	69.33

**Table 2:** Effect of vaccinations for COVID-19 and influenza outside pregnancy on vaccination during pregnancy (age-adjusted) (n = 375).

Vaccination Outside Pregnancy	OR	95%CI	*p*-Value
COVID-19 vaccination	4.61	2.60–8.22	<0.001 *
Influenza vaccination	7.14	3.58–14.25	<0.001 *

* *p* < 0.05.

**Table 3:** Maternal vaccination use and study participants’ education (n = 375).

Maternal Vaccinations Used in the Latest Pregnancy	All Study Participants (n = 375)	Non-Healthcare Education (n = 259)	Healthcare Education (n = 116)	*p* (chi^)
Yes	208 (55.47%)	149 (57.53%)	59 (50.86%)	*p* = 0.230 1.442
No	167 (44.53%)	110 (42.47%)	57 (49.14%)	

**Table 4:** Vaccination attitudes and use of maternal vaccine prevention (n = 375).

Relevant Item of the VAX Scale	All Study Participants (n = 375)	Use of Maternal Vaccinations (n = 208)	Non-Use of Maternal Vaccinations (n = 167)	Z	*p*-Value
Mistrust of Vaccine Benefit	7.71 ± 4.40 6 (3–18)	6.02 ± 3.46 5.5 (3–18)	9.82 ± 4.54 11 (3–18)	9.068	<0.001 *
Worries over Unforeseen Future Effects	10.90 ± 4.12 11 (3–18)	10.90 ± 4.17 11 (3–18)	10.91 ± 4.06 11 (3–18)	−0.097	0.923
Concerns about Commercial Profiteering	6.58 ± 3.68 6 (3–18)	5.17 ± 2.55 5 (3–18)	8.34 ± 4.09 8 (3–18)	8.100	<0.001 *
Preference for Natural Immunity	7.49 ± 3.65 7 (3–18)	6.50 ± 2.83 6 (3–16)	8.72 ± 4.16 8 (3–18)	5.219	<0.001 *

* *p* < 0.05.

**Table 5:** Influence of maternal vaccination on respondents’ attitudes (age-adjusted) (n = 375).

Model	b	Standard Error	T	95% Conf. Interval		*p*-Value
Model 1: Mistrust of Vaccine Benefit						
	*p* < 0.001, R-squared = 0.183, MSE = 10.43					
Maternal vaccination	−3.77	0.41	−9.11	−4.59	−2.96	<0.001 *
Model 2: Worries over Unforeseen Future Effects						
	*p* = 0.006, R-squared = 0.027, MSE = 11.058					
Maternal vaccination	−0.13	0.42	−0.32	−0.97	0.70	0.751
Model 3: Concerns about Commercial Profiteering						
	*p* < 0.001, R-squared = 0.190, MSE = 7.335					
Maternal vaccination	−3.22	0.35	−9.31	−3.90	−2.54	<0.001 *
Model 4: Preference for Natural Immunity						
	*p* < 0.001, R-squared = 0.098, MSE = 8.064					
Maternal vaccination	−2.28	0.36	−6.28	−2.99	−1.57	<0.001 *

* *p* < 0.05.

**Table 6:** Influence of specific maternal vaccinations on respondents’ attitudes (age-adjusted) (n = 375).

Model	b	Standard Error	T	95% Conf. Interval		*p*-Value
Model 1: Mistrust of Vaccine Benefit						
	*p* < 0.001 *, R-squared = 0.186, MSE = 12.718					
Diphtheria, tetanus and pertussis	−2.57	0.46	−5.58	−3.47	−1.66	<0.001 *
Seasonal influenza	−1.75	0.57	−3,05	−2.88	−0.62	0.002 *
COVID-19 (SARS-CoV-2)	−1.14	0.50	−2.28	−2.12	−0.16	0.023 *
Model 2: Worries over Unforeseen Future Effects						
	*p* < 0.001 *, R-squared = 0.060, MSE = 12.888					
Diphtheria, tetanus and pertussis	−0.89	0.46	−1.92	−1.80	0.02	0.055
Seasonal influenza	−0.13	0.58	−0.22	−1.26	1.01	0.826
COVID-19 (SARS-CoV-2)	1.65	0.50	3.30	0.67	2.64	0.001 *
Model 3: Concerns about Commercial Profiteering						
	*p* < 0.001 *, R-squared = 0.194, MSE = 8.801					
Diphtheria, tetanus and pertussis	−1.63	0.38	−4.26	−2.38	−0.88	<0.001 *
Seasonal influenza	−1.61	0.48	−3.38	−2.55	−0.67	0.001 *
COVID-19 (SARS-CoV-2)	−1.69	0.41	−4.09	−2.51	−0.88	<0.001 *
Model 4: Preference for Natural Immunity						
	*p* < 0.001 *, R-squared = 0.115, MSE = 9.542					
Diphtheria, tetanus and pertussis	−1.37	0.40	−3.45	−2.16	−0.59	0.001 *
Seasonal influenza	−0.68	0.50	−1.36	−2.99	−1.57	0.173
COVID-19 (SARS-CoV-2)	−1.60	0.43	−3.70	−2.45	−0.75	<0.001 *

* *p* < 0.05.

**Table 7:** Reasons for being vaccinated during pregnancy (n = 208) *.

Reasons for Being Vaccinated during Pregnancy	N	%
Concern for the health and safety of the unborn child	196	94.23
Concern for one’s own health and safety	139	66.83
Recommendations from the pregnant woman’s healthcare provider	135	64.90
Positive experiences related to vaccinations	40	19.23
Risk of infection with contagious diseases from my older children	23	11.06
Positive opinions of family/friends about maternal vaccinations	20	9.62
Risk of infection with contagious diseases in the workplace	19	9.13
Positive opinions about maternal vaccinations in the media	16	7.69
Foreign travel requirement	3	1.44

* The respondents could indicate more than one answer.

**Table 8:** Reasons for not being vaccinated during pregnancy (n = 167) *.

Reasons for Not Being Vaccinated during Pregnancy	n	%
No recommendations from the pregnant woman’s healthcare provider	103	61.68
Fear for the health and safety of the unborn child	88	52.69
Fear for one’s own health and safety	51	30.54
Negative opinions of family/friends about maternal vaccinations	12	7.19
No financial resources to purchase vaccines	5	2.99
Negative opinions about maternal vaccinations in the media	4	2.40
Medical contraindications	1	0.60

* The respondents could indicate more than one answer.

## Data Availability

The original contributions presented in the study are included in the article, further inquiries can be directed to the corresponding author.

## Appendix A

**Table A1:** Adult vaccinations (outside the pregnancy period) and study participants’ education level (n = 375).

Adult Vaccinations	All Study Participants (n = 375)	Non-Healthcare Education (n = 259)	Healthcare Education (n = 116)	*p* (chi^)
Influenza vaccination (in the last five years)				
Yes	89 (23.73%)	61 (23.55%)	28 (24.14%)	*p* = 0.902 chi^ = 0.015
No	286 (76.27%)	198 (76.45%)	88 (75.86%)	
COVID-19 vaccination				
Yes	281 (74.93%)	196 (75.68%)	85 (73.28%)	*p* = 0.620 chi^ = 0.246
No	94 (25.06%)	63 (24.32%)	31 (26.72%)	
